# Retraction: Epigenetic Regulation of Cardiac Progenitor Cells Marker c-kit by Stromal Cell Derived Factor-1α

**DOI:** 10.1371/journal.pone.0247094

**Published:** 2021-02-12

**Authors:** The PLOS ONE Editors

Following the publication of this article [1], concerns were raised regarding similarity between panels presented in Fig 3C, as well as similarity between figures presented in this article [1] and figures presented in a publication accepted in the journal *Heart*, *Lung and Circulation* [2] prior to the acceptance of this article [1] in *PLOS ONE*. In addition, concerns were raised regarding substantial overlap with the work reported in both articles [1, 2]. Specifically,

- The panels in Fig 1A and 1B [1] appear similar to the panels in Fig 1B and 1C [2] respectively.
- The c-kit(+)CPCs control panel in Fig 3C [1] partially overlaps with the c-kit(+)CPCs SDF-1+AMD3100 panel in Fig 3C [1], the normal panel of Fig 3C [2], and the SDF-1+AMD3100 panel of Fig 3C [2].
- There is overlap in the experimental design, results and analyses between the *PLOS ONE* article [1] and those reported in [2]. The related paper is not cited in the *PLOS ONE* article and was not disclosed at the time of submission.

The authors have provided underlying data for the results presented in Fig 3 and indicated that the two Fig 3C panels [1] appear similar because the wrong image was included in the figure. The authors indicate that the experimental data in this article [1] appears similar to the data presented in [2] because several experiments were repeated, and they state that experimental data was not reused for both articles. However, the authors’ statement that the experimental data was not reused does not sufficiently address concerns regarding the similarity between multiple panels presented in these articles.

In light of the concerns about redundancy of this work, the *PLOS ONE* Editors retract this article.

The panels of Figs 1A, 1B and 3C listed above appear visually similar to copyright protected material which is not offered under a CC-BY licence. The indicated panels therefore are excluded from the PLOS ONE article’s [1] licence. At the time of retraction, the article [1] was republished to note this exclusion in the Figs 1 and 3 legends and the article’s copyright statement.

ZC agreed with the retraction and apologises for the issues with the published article. XP, YY, FY, LC, RH, and GM either did not respond directly or could not be reached.
